# The complete mitochondrial genome of the mudskipper, *Boleophthalmus pectinirostris* (Gobiiformes, Oxudercidae) from Beibu Bay

**DOI:** 10.1080/23802359.2021.1909433

**Published:** 2021-04-05

**Authors:** Chuanyan Pan, Shan Xiao, Xiuli Chen, Chunling Yang, Digang Zeng, Pengfei Feng, Min Peng

**Affiliations:** a Guangxi Key Laboratory of Aquatic Genetic Breeding and Healthy Aquaculture, Nanning, China; b Guangxi Academy of Fishery Sciences, Nanning, China; c Guangxi Aquatic Species Introduction & Breeding Center, Nanning, China

**Keywords:** *Boleophthalmus pectinirostris*, mitochondrial genome, Oxudercidae

## Abstract

The mudskipper, *Boleophthalmus pectinirostris* (*B. pectinirostris*), is an amphibious fish that lives in the intertidal mudflats. It is a cultured economic fish with nutritional and pharmacological value. Here, we report the complete mitochondrial genome sequence of *B. pectinirostris*, which is 17,111 base pairs (55.3% A + T content) in length and consists of 13 protein-coding genes, 22 transfer RNAs, 2 ribosomal RNAs, and a 1453 bp D-loop region. The complete mitochondrial genome of *B. pectinirostris* will provide useful genetic information for future phylogenetic and taxonomic classification of *B. pectinirostris*.

The mudskipper, *Boleophthalmus pectinirostris* (Gobiiformes, Oxudercidae) is an amphibious gobioid teleosts, which is a commercially important aquaculture species (Zhao et al. 2018). It is a warm water fish that distributes in tropical mudflats along the coast of Japan, Taiwan and Southern China (Clayton 1993; Liu et al. 2012). *B. pectinirostris*lives in the intertidal zone along the coastland estuarine areas where the salinity change is intense, hence it shows salinity tolerance (Chen et al. 2006). The physiological, biochemical and adaptive mechanisms of *B. pectinirostris* have become hot research issues, due to its habitat specificity and unique amphibious adaptability. Natural populations of *B. pectinirostris* are gradually decreasing due to the overfishing and habitat destruction (Zhang and Hong 2006). Mitochondrial DNA (mtDNA) is particularly suitable for evolutionary studies because it has the unique characteristics of strict maternal inheritance, high copy number, and rapid evolution (Stoneking and Soodyall 1996). The complete mitochondrial genome is an excellent molecular marker for studying phylogenetic relationships and species identification (Zhong et al. 2020). Here, we report the complete mitochondrial genome of *B. pectinirostris* and the analyses of the mitogenomic phylogenetic. The results of this study will provide reference for genetics research of *B. pectinirostris*.

The *B. pectinirostris* samples were collected from Hepu (21.593357 N, 109.087536E), Beihai, Guangxi, China. The whole body specimens (#BH201905310001) were deposited at Guangxi Key Laboratory of Aquatic Genetic Breeding and Healthy Aquaculture, Nanning, China. The total genomic DNA was extracted from the fins of one *B. pectinirostris* samplevia the phenolchloroform extraction method (Kumar and Mugunthan 2018). DNA libraries (350 bp insert) were constructed using the TruSeq NanoTM kit (Illumina, San Diego, CA) and were sequenced (2 × 150bp paired-end) on a HiSeq platform by Novogene Company, China. Sequence assembly was performed using the MITObim software (Hahn et al. 2013). Gene annotation was performed using the MITOS software (http://mitos2.bioinf.uni-leipzig.de/). The phylogenetic tree was constructed using maximum-likelihood method.

The complete mitochondrial genome of *B. pectinirostris* is 17,111 bp in length (GenBank accession number: MN909967) with the base composition of A (25.7%), T (29.6%), C (29.0%) and G (15.7%). The percentage of G + C is 44.7%, and the percentage of A + T is 55.3%, which is similar to the giant mudskipper, *Periophthalmodon schlosseri* (42.4% of G + C and 57.6% of A + T) (Yi et al. 2016). The mitochondrial genome of *B. pectinirostris* contains 13 protein-coding genes, 22 transfer RNAs, 2 ribosomal RNAs (a 12S rRNA and a 16S rRNA), and a D-loop region.

The mitogenomic phylogenetic analyses showed that *B. pectinirostris* was first clustered with *B. pectinirostris* collected from Cixi, China (NC 016195.1), whose complete mitochondrial genome is 17,111 bp in length, base composition is 29.7% for A, 28.3% for C, 26.6% for T and 15.4% for G, which are similar to those of the *B. pectinirostris* in this study, but the content of A + T (56.3%) is higher (55.3% in this study). Then, *B. pectinirostris* was clustered with *Boleophthalmus boddarti* (*B. boddarti*) (Figure 1), indicating the close relationship between *B. pectinirostris* and *B. boddarti*The complete mitochondrial genome sequence of *B. pectinirostris* will enrich the genome data of Gobiiformes, and will be useful for taxonomy research, conservation, and management.

**Figure 1.**
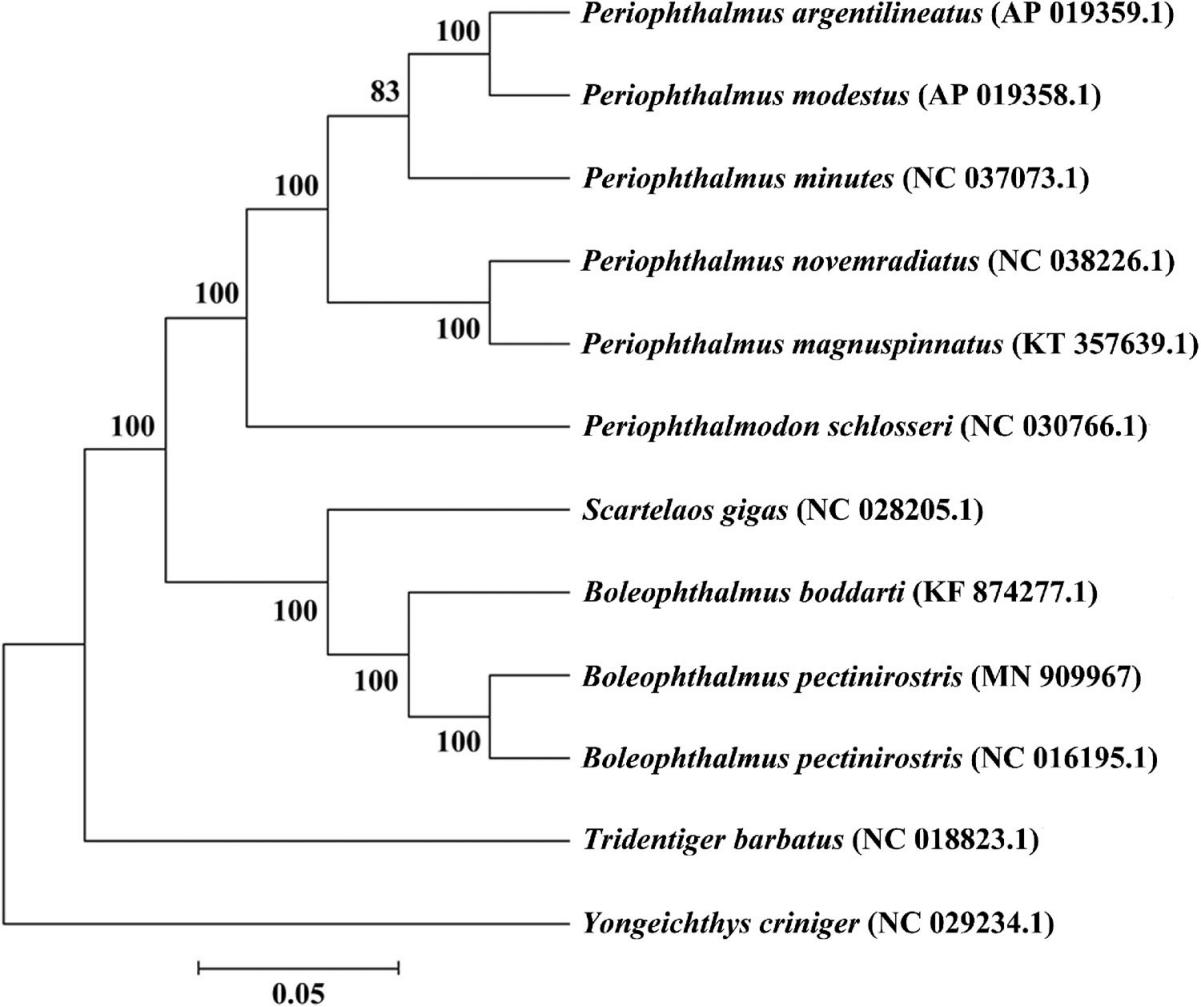
A phylogenetic tree was constructed based on the comparison of mitochondrial genome sequences of B. pectinirostris and other species of Gobiidae. All the sequences were downloaded from NCBI GenBank.

## Data Availability

The data that support the findings of this study are openly available in National Center for Biotechnology Information (https://www.ncbi.nlm.nih.gov) under the accession MN909967. The associated BioProject, SRA, and BioSample numbers are PRJNA688752, SRR13334530, and SAMN17188496822, respectively.

## References

[CIT0001] Chen SX, Hong WS, Zhang QY, Wu RX, Wang Q. 2006. Rates of oxygen consumption and tolerance of hypoxia and desiccation in Chinese black sleeper (*Bostrichthys sinensis*) and mudskipper (*Boleophthalmus pectinirostris*) embryos. Acta Oceanolog Sin. 25(4):91–98.

[CIT0002] Clayton DA. 1993. Mudskippers. Oceanogr Mar Biol: An Annu Rev. 37:507–577.

[CIT0003] Hahn C, Bachmann L, Chevreux B. 2013. Reconstructing mitochondrial genomes directly from genomic next-generation sequencing reads-a baiting and iterative mapping approach. Nucleic Acids Res. 41(13):e129–e129. 23661685 10.1093/nar/gkt371 PMC3711436

[CIT0004] Kumar M, Mugunthan M. 2018. Evaluation of three DNA extraction methods from fungal cultures. Med J Armed Forces India. 74(4):333–336. 30449918 10.1016/j.mjafi.2017.07.009 PMC6224647

[CIT0005] Liu ZZ, Wang CT, Ma LB, He AY, Yang JQ, Tang WQ. 2012. Complete mitochondrial genome of the mudskipper *Boleophthalmus pectinirostris* (Perciformes, Gobiidae): Repetitive sequences in the control region. Mitochondrial DNA. 23(1):31–33. 22295864 10.3109/19401736.2011.643879

[CIT0006] Stoneking M, Soodyall H. 1996. Human evolution and the mitochondrial genome. Curr Opin Genet Dev. 6(6):731–736. 8994844 10.1016/s0959-437x(96)80028-1

[CIT0007] Yi YH, Zhang K, Chen JM, Ruan ZQ, You XX, Shi Q. 2016. The complete mitochondrial genome sequence of the giant mudskipper, *Periophthalmodon schlosseri* (Perciformes: gobiidae). Mitochondrial DNA B Resour. 1(1):599–600. 33490412 10.1080/23802359.2016.1202742 PMC7800980

[CIT0008] Zhang QY, Hong WS. 2006. Review and prospect of mudskipper *Boleophthalmus pectinirostris* studies. J Xiamen Univ (Nat Sci). 45(2):97–108.

[CIT0009] Zhao YQ, Mu DL, Wang D, Han YL, Hou CC, Zhu JQ. 2018. Analysis of the function of kif3a and kif3b in the spermatogenesis in *Boleophthalmus pectinirostris*. Fish Physiol Biochem. 44(3):769–788. 29511984 10.1007/s10695-017-0461-1

[CIT0010] Zhong SP, Huang LH, Liu YH, Huang GQ, Chen XL. 2020. The complete mitochondrial genome of *Phascolosoma similis* (Sipuncula, Phascolosomatidae) from Beibu Bay. Mitochondrial DNA Part B. 5(2):1263–1264.

